# Patient‐specific 17‐segment myocardial modeling on a bull's‐eye map

**DOI:** 10.1120/jacmp.v17i5.6237

**Published:** 2016-09-08

**Authors:** Joonho Jung, Young‐Hak Kim, Namkug Kim, Dong Hyun Yang

**Affiliations:** ^1^ Departments of Cardiology University of Ulsan College of Medicine Seoul Korea; ^2^ Convergence Medicine, University of Ulsan College of Medicine Seoul Korea; ^3^ Radiology, University of Ulsan College of Medicine Seoul Korea

**Keywords:** patient‐specific, 17‐segment myocardial model, bull's‐eye map, severe aortic stenosis

## Abstract

The purpose of this study was to develop and validate cardiac computed tomography (CT) quantitative analysis software with a patient‐specific, 17‐segment myocardial model that uses electrocardiogram (ECG)‐gated cardiac CT images to differentiate between normal controls and severe aortic stenosis (AS) patients. ECG‐gated cardiac CT images from 35 normal controls and 144 AS patients were semiautomatically segmented to create a patient‐specific, 17‐segment myocardial model. Two experts then manually determined the anterior and posterior interventricular grooves to be boundaries between the 1st and 2nd segments and between the 3rd and 4th segments, respectively, to correct the model. Each segment was automatically identified as follows. The outer angle of two boundaries was divided to differentiate the 1st, 4th, 5th, and 6th segments in the basal plane, whereas the inner angle divided the 2nd and 3rd segments. The segments of the midplane were similarly divided. Segmental area distributions were quantitatively evaluated on the bull's‐eye map on the basis of the morphological boundaries by measuring the area of each segment. Segmental areas of severe AS patients and normal controls were significantly different (t‐test, all p‐values<0.011) in the proposed model because the septal regions of the severe AS patients were smaller than those of normal controls and the difference was enough to divide the two groups. The capabilities of the 2D segmental areas (p<0.011) may be equivalent to those of 3D segmental analysis (all p‐values<0.001) for differentiating the two groups (t‐test, all p‐values<0.001). The proposed method is superior to the conventional 17‐segment in relation to reflection of patient‐specific morphological variation and allows to obtain a more precise mapping between segments and the AHA recommended nomenclature. It can be used to differentiate severer AS patients and normal controls and also helps to understand the left ventricular morphology at a glance.

PACS number(s): 87.57.N‐, 87.57.R‐, 87.57.qp

## I. INTRODUCTION

The bull's‐eye map, a polar plot from tomographic images of the left ventricle (LV), has been used for regional analysis of LV function or myocardial perfusion for more than 20 years.^(1)^ This map displays information on the whole LV in a single image, including the relationship between a myocardial segment and its supplying coronary arteries. The latest standardization of the bull's‐eye map, the standardized 17‐segment model, was published by the American Heart

Association (AHA) in 2002^(2)^ and is currently considered the conventional standard. However, the AHA noted that the name of the segments should define the location relative to the long axis of the heart and the circumferential location, and individual myocardial segments can be assigned to the three major coronaries by considering their anatomic variabilities.^(2)^ In addition, contrary to the conventional 17‐segment model, several studies have reported, using single‐photon emission computed tomography (SPECT), cardiac magnetic resonance (CMR), and CT, that there are variations in the mapping of segments to left ventricular territories, due to their anatomic variations.^3^, ^4^, ^5^ Therefore, they proposed a revised mapping of segments considering anatomic variations, where some segments were assigned to multiple territories due to per‐patient variability. Therefore, there is a need for a better bull's‐eye map than that of the conventional 17‐segment model that could more accurately reflect patient‐specific anatomic variations.

For the bull's‐eye map, Cain et al.^(6)^ presented a procedure for a quantitative polar representation of LV myocardial perfusion, function, and viability using SPECT and CMR. They described how to generate polar plots using quantitative data based on the conventional 17‐segment model. They treated all data as polar coordinates (slice number as radius, segment position as angle) and subsequently transformed the coordinates for graphing as polar plots. Oeltze et al.^(7)^ reported several novel visualization techniques that enabled the myocardial perfusion data of both rest and stress states to be shown in a single bull's‐eye plot based on the 17‐segment model. Their model involved an interactive link between their bull's‐eye map and a 3D visualization showing the coronary artery branches. However, this study did not consider patient‐specific anatomic variations in their bull's‐eye plot.

There have been several studies of patient‐specific bull's‐eye maps of the LV.^8^, ^9^, ^10^, ^11^ These studies reported patient‐specific mappings between each of the 17 segments and LV territories using CMR, adapting the 17‐segment model using patient‐specific anatomy. The studies' approach achieved better correspondence between the 17 segments and coronary arterial territories, but could not provide a precise mapping between segments and the AHA recommended nomenclature. In fact, these two approaches are completely different concepts and cannot therefore be satisfied at the same time. Thus, a new approach is needed to precisely divide the LV myocardium into 17 segments according to the AHA recommended nomenclature.

The aim of the present study was to propose a novel 17‐segment myocardial model that considered the patient‐specific morphological variations in LV anatomy to obtain a more precise mapping between segments and the AHA recommended nomenclature. The details are presented in sections II‐IV. In section II, material and methods are introduced, and the experimental results are described in detail in section III. Finally, the discussion and conclusion of our approach are reported in sections IV and V.

## II. MATERIALS AND METHODS

**Figure 1:**
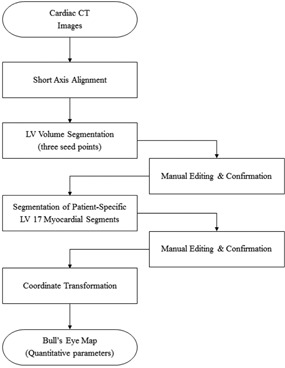
Schematic depiction of the steps in the proposed cardiac CT analysis method.

In our method, short‐axis alignment was performed first after which the LV volume was segmented by seeded region growing method and modeled by finding appropriate control points to represent the inner and outer boundaries of the segmented LV volume. The patient‐specific, 17‐myocardial segments were modeled by delineating the myocardial septum boundaries using the anterior and posterior interventricular grooves. And these boundaries were projected onto the bull's‐eye map. The area of each segment on the bull's‐eye map was measured and the ability of the segmental area to differentiate between normal controls and severe aortic stenosis (AS) patients was evaluated. Figure 1 shows a flow chart that schematically depicts this method.

### A. Short‐axis alignment

The short‐axis view shows cross‐sections of the LV that are useful for volumetric measurements. This view was chosen so that the series of slices would be perpendicular to the long axis of the LV that transects the apex and the center of the mitral valve plane.^(2)^ The first step involved finding the center of the mitral valve and the apex point to show the correct short‐axis view. The mitral valve was seen in the axial view and the center of the mitral valve was chosen by adjusting the location of the slice through the axial and sagittal views. The cardiac volume was rotated on the center of the mitral valve and showed a roughly defined short axial view. To achieve a more errorless short‐axis view, the cardiac volume was rerotated on the recalculated long axis defined by the center of the mitral valve and the apex point that were confirmed in section C below.

### B. Left ventricular segmentation

The LV volume segmentation was performed using a thresholding method. From the three seed points (SP1, SP2, and SP3), a seeded region growing method was performed to search for 3D connected regions. The SP1 was the center point between the apex and endo‐apex. The SP2 was the center of the long axis that was the center point between the apex and the center of the mitral valve. It was used for detecting the largest connected component that represented the chamber volume of the LV The SP3 was the center of the mitral valve. It was used for the upper boundary of the LV, whereas the apex point was used for the lower boundary. After selecting the seed points, we searched for the largest connected component that represented the LV volume with a specific size by moving from the bottom slice to the top slice.

The inner and outer boundaries of the LV (i.e., epi‐ and endomyocardial borders) were fitted to the surface by two radiographers with more than five years of experience each. To reduce the manual burden and improve reliability, our quantitative cardiac analysis software platform provided an initial surface contour for each slice. It was modeled using a closed spline curve, consisted of 10 control points and the initial shape was a circle. The position of each control point was calculated from the minimum and maximum boundaries of the generated connected component on the axial view. Two experts modified the initially obtained surface contours, and a cardiac radiologist with more than 10 years of experience confirmed the LV surface contour and its validity.

**Figure 2:**
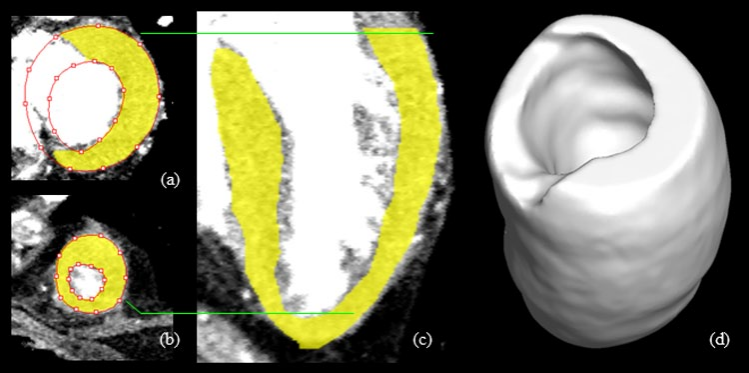
Overlay of the left ventricular segmentation (yellow) on CT: ((a), (b)) LV surface contour overlaid on axial sections with differences indicated (green lines indicate the level on the coronal section); (c) segmented LV mask overlaid on a coronal section; (d) 3D visualization of the LV.

An LV volume mask was generated from the LV surface contour. In theory, this mask could include some part of the aortic root. Therefore, we removed the aortic root from the LV volume by using a thresholding method and basic morphological operation.^12^, ^13^ The aortic root was extracted as a region where the pixel intensities were higher than the value of 200 HU between the basal anteroseptal and inferoseptal regions. The morphological “dilate” and “erode” operations were used to include the aortic valve into the aortic root because it has a lower intensity value than 200 HU. The aortic root‐excluded LV volume was finally corrected and confirmed by the experts. Figure 2 shows the result of LV segmentation.

### C. Segmentation of patient‐specific LV 17 myocardial segments

**Figure 3:**
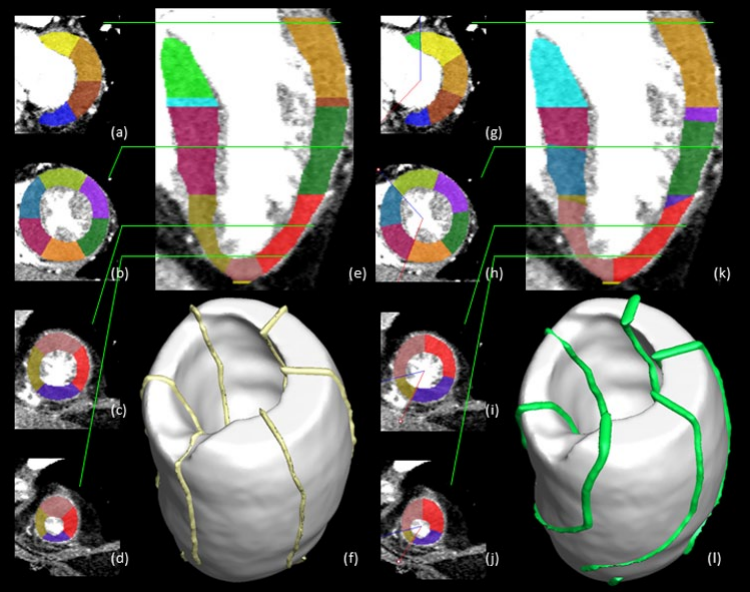
Comparison view of the conventional ((a)–(f)) and patient‐specific ((g)–(l)) 17‐segment models. The conventional 17‐segment model ((a)–(d)) overlaid on an axial section with differences indicated (green lines indicate the level on the coronal image (e). The patient‐specific 17‐segment model ((g)–(j)) overlaid on an axial section with differences indicated (green lines indicate the level on the coronal image (k)). 3D visualization ((f),(l)) of the conventional and patient‐specific 17 segments models, respectively.

The segmented LV volume was divided into newly defined patient‐specific 17 myocardial segments. The conventional model divided the myocardium into six segments of 60° each on the basal and midcavity slices in Figs. 3(a) to 3(f).^(2)^ However, the proposed model divided the 17 myocardial segments on the basis of geometry landmarks in Figs. 3(g) to 3(l). The LV septum region was semiautomatically segmented and mapped to five septal segments: the basal anteroseptal, basal inferoseptal, midanteroseptal, midinferoseptal, and apical septal segments (i.e., the 2nd, 3rd, 8th, 9th, and 14th segments, respectively). The anterior and posterior interventricular grooves were manually divided by setting boundaries between the 1st and 2nd segments and between the 3rd and 4th segments, respectively, on the basal plane. Other segments in circumferential locations were automatically divided based on the septum region as follows. The outer angle of two boundaries was divided into the 1st, 4th, 5th, and 6th segments in the basal plane, whereas the inner angle was divided into the 2nd and 3rd segments. Similarly, the midcavity plane was split into six segments. In the apical plane, the 14th segment was divided by the anterior and posterior interventricular grooves. The outer angle of the two boundaries was divided to differentiate the 13th, 15th, and 16th segments of the apical plane. It takes 5 to 10 min to divide LV septum region by moving the blue line to the anterior interventricular grooves and the red line to posterior interventricular grooves in each slice (Figs. 3(g) to 3(j)). Finally, a cardiac radiologist manually corrected and confirmed proposed boundaries of the LV septum region.

### D. Patient‐specific bull's‐eye maps

**Figure 4:**
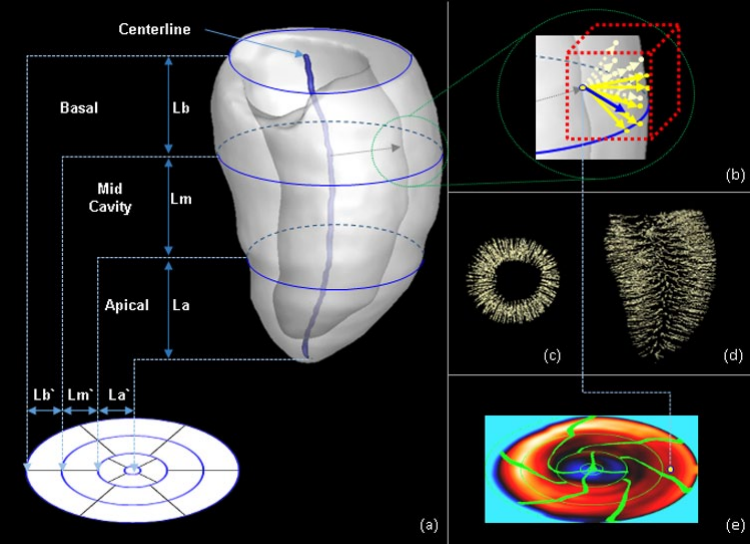
Mapping of the LV myocardium to a bull's‐eye plot and measuring thickness: (a) projection method for 3D LV myocardium onto a two‐dimensional bull's‐eye plot; (b) method for measuring thickness; (c) result of measuring thickness on coronal view; (d) result of measuring thickness on axial view; (e) color‐coded bull's‐eye map with result of thickness measurement.

The bull's‐eye map is a quantitative polar representation of LV volume.^(1)^ It helps physicians to recognize various quantitative parameters of the LV at a glance, such as the intensity, intensity difference, and transmural perfusion ratio and thickness.^14^, ^15^, ^16^, ^17^, ^18^, ^19^, ^20^, ^21^, ^22^ This 2D polar plot was generated from 17 myocardial segments on 3D polar coordinates. All short‐axis slices of the LV volume were converted into a series of rings (Fig. 4(a)) by measuring a quantitative parameter in each pixel lying on a radius between the inner and outer boundaries and the radius was rotated on the center of LV myocardium for all short‐axis slices (Fig. 4(b)).^(6)^ The radius of each ring grew when moving toward the basal of the heart from the apex. The maximum radius of the bull's‐eye plot was fixed while the distance from the apex to the top of basal varies from patient to patient so the radius that represented the proportional distance from the apex to that short‐axis view (Fig 4(a)). The second circle from the outermost circle was used for the boundary between the basal and midcavity regions, whereas the third circle was used for the boundary between the midcavity and apical regions. The position of these rings was not changed for the patient‐specific 17 myocardial segments. The radii of these two circles were one‐third and two‐thirds of the maximum radius, respectively. The center of the myocardium was calculated by averaging the center of the epimyocardial and endomyocardial borders. The center of the epicardium was treated as the center of the myocardium at the apex region. Figure 4(a) shows a blue colored centerline calculated with proposed method. The thickness of the myocardium was measured by finding the closest point on the epimyocardial from a point on endomyocardial

(Fig. 4(b)). Figures 4(c) and 4(d) sparsely visualized measured data on coronal view and axial view, respectively. Finally, the measurement result of each point on endomyocardial was color‐coded on the bull's‐eye map (Fig. 4(e)).

**Figure 5:**
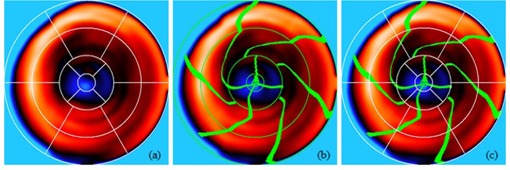
Conventional bull's‐eye map vs. patient‐specific bull's‐eye map. The thickness of the LV was plotted on the bull's‐eye map. (a) Conventional bull's‐eye map. (b) Patient‐specific bull's‐eye map. (c) Overlaid bull's‐eye map.

Figure 5(a) shows the conventional 17 myocardial segments. This model divided the basal and midcavity slices into six segments of 60° each and the apical slices into four segments of 90° each.^(2)^

Figure 5(b) shows the proposed 17 myocardial segments. The morphological boundaries of the proposed 17‐segment model were projected onto the bull's‐eye map. The two lines at 11 o'clock and 7 o'clock corresponded to the anterior interventricular groove and posterior interventricular groove, respectively. Each segment on the same circumferential location on Fig. 5(c) has different room size. Thus, we quantitatively measured the occupied space of each segment by counting the number of pixels inside the boundary of each segment on the bull's‐eye map. However, the number of pixels is too big and not a physical unit and it is difficult to intuitively map between each segment and the numbers. Hence, we multiply the dot pitch of the monitor (0.2692 mm, Dell U2412M) (Dell, Inc., Round Rock, TX) to the number of pixels and use this value as the area of each segment. The segments' area has a physical unit (cm^2^) and it is easy to map between each segment and the numbers at a glance.

### E. Validation and statistical analysis

To validate the usability of the patient‐specific, 17‐segment myocardial model, 2D quantitative parameters were evaluated from two 17‐segment models on the bull's‐eye map and the 3D parameters were evaluated from volume data. We evaluated whether these parameters could differentiate between normal controls and severe AS patients on the basis of the clinical knowledge that the LV response to aortic stenosis includes LV remodeling.^23^, ^24^, ^25^, ^26^, ^27^, ^28^ MATLAB R2013a (MathWorks, Natick, MA) was used to perform paired *t*‐tests and a diagnostic classification test.

## III. EXPERIMENTAL RESULTS

### A. Subjects and dataset

A retrospective study was performed to validate our patient‐specific, 17‐segment myocardial model. Thus, we used already clinically differentiated groups including AS patients and normal controls that had different anatomic variation. In total, 179 patients underwent electrocardiogram‐gated volumetric cardiac CT scans in the Department of Radiology, Asan Medical Center, South Korea; 144 patients had severe AS and 35 patients had a normal cardiac anatomy. The CT scans were obtained by a second‐generation, dual‐source CT scanner (Definition Flash; Siemens, Forchheim, Germany) with a smooth kernel (B26f) and 0.64‐mm slice thickness. The conventional and patient‐specific 17‐segment models were semiautomatically modeled, as described in the Materials and Methods sections A to D. The thickness and mass of each segment in the 3D model and the area of each segment on the bull's‐eye map was quantitatively assessed considering the morphological boundaries. In‐house software was used to measure these parameters. We performed paired *t*‐test to evaluate the independence of quantitative parameters from two groups using the area of each segment and a diagnostic classification test to evaluate whether these parameters could differentiate between normal controls and severe AS patients using the maximum likelihood that was estimated from the area of each segment. The proposed method required a manual adjustment to divide the segmented LV volume into the patient‐specific 17 myocardial segments. Therefore, we compared interoperator and intraoperator variability for this procedure using data from 10 randomly selected patients.

### B. Results

**Figure 6:**
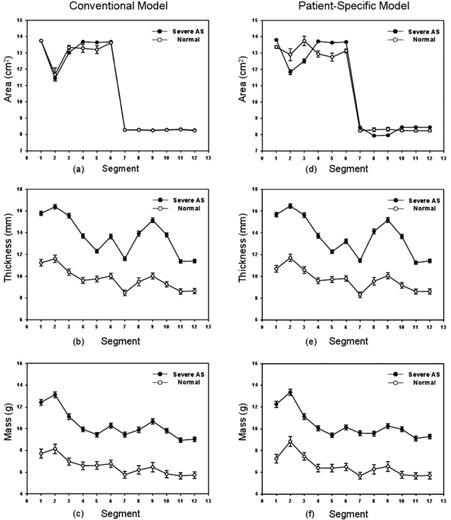
Segmental distribution of quantitative parameters. The mean and standard error of each parameter were plotted. The segmental area distribution ((a), (d)) for the two models. The segmental maximum thickness ((b), (e)). The mass of each segment ((c), (f)).

We evaluated the ability of the 3D and 2D parameters of the two 17‐segment models to differentiate between normal controls and severe AS patients. The differences between the two 17‐segment models were described in the Materials & Methods section C above. The evaluation results are shown in Table 1 and Table 2. Figure 6 shows the segmental distributions of the quantitative parameters. The 3D parameters of the two 17‐segment models were significantly different (p<0.001) between severe AS patients and normal controls. In the case of the 2D parameters, the two models were completely different. Most segments of the conventional model, except segment 3 (p=0.035), showed no differences (p>0.073) between the two groups. However, the parameters of all segments of the proposed model were significantly different (p<0.011).

The evaluation results in Table 1 were similar to the segmental distribution (Fig. 6). Each segmental value of the 3D parameters of the two groups did not overlap and were dividable (Figs. 6(b), 6(c), 6(e), 6(f)). For the 2D parameters, the areas of segments 1 and 6 and that of all midcavity segments of the conventional model were almost the same (Fig. 6(a)). From these two results, the capabilities of the 2D segmental areas (p<0.011) may be equivalent to those of 3D segment analysis (all p‐values<0.001) for differentiating the two groups.

Table 2 presents the sensitivity, specificity, and area under curve (AUC) of a diagnostic classification test. The evaluation results showed a tendency toward a higher specificity value, higher AUC value, and lower sensitivity value for the proposed model in comparison to the conventional model. The mean value of specificity for the proposed model (0.59±0.19) was more than twice that of the conventional model (0.27±0.06). The AUC for the proposed model (0.69±0.05) was bigger than that of the conventional model (0.60±0.12). Especially, the specificity and AUC for segment 1 of the proposed model (0.71 and 0.72) were more than double that of the conventional model (0.26 and 0.27).

**Table 1:** Capability of the two models to differentiate between normal controls and severe aortic stenosis patients. Data are reported as mean values.

	*Conventional Model*									*Proposed Model*								
	*3D*						*2D*			*3D*						*2D*		
	*Thickness (mm)*			*Mass (g)*			*Area (cm^2^)*			*Thickness (mm)*			*Mass (g)*			*Area (cm^2^)*		
*Segment*	*AS*	*Nor*.	p‐*value*	*AS*	*Nor*.	p‐*value*	*AS*	*Nor*.	p‐*value*	*AS*	*Nor*. p‐*value*		*AS*	*Nor*.	p‐*value*		*Nor*.	p‐*value*
Seg.1	15.8	11.2	^a^	12.4	7.7	^a^	13.7	13.7	0.922	15.7	10.7	^a^	12.2	7.2	^a^	13.8	13.4	^a^
Seg.2	16.4	11.6	^a^	13.1	8.1	^a^	11.4	11.7	0.589	16.5	11.7	^a^	13.4	8.8	^a^	11.8	12.9	^c^
Seg.3	15.6	10.4	^a^	11.1	7.0	^a^	13.0	13.3	^c^	15.6	10.6	^a^	11.1	7.5	^a^	12.5	13.7	^a^
Seg.4	13.7	9.6	^a^	9.9	6.6	^a^	13.7	13.3	0.114	13.7	9.6	^a^	10.0	6.4	^a^	13.7	12.9	^a^
Seg.5	12.3	9.7	^a^	9.5	6.6	^a^	13.6	13.2	0.077	12.3	9.7	^a^	9.4	6.4	^a^	13.6	12.8	^a^
Seg.6	13.7	10.0	^a^	10.3	6.8	^a^	13.7	13.6	0.350	13.2	9.8	^a^	10.1	6.5	^a^	13.7	13.1	^a^
Seg.7	11.6	8.5	^a^	9.4	5.8	^a^	8.3	8.3	0.962	11.5	8.3	^a^	9.6	5.7	^a^	8.4	8.2	^b^
Seg.8	13.9	9.5	^a^	9.9	6.2	^a^	8.3	8.3	0.409	14.1	9.6	^a^	9.6	6.3	^a^	7.9	8.3	^b^
Seg.9	15.1	10.0	^a^	10.7	6.5	^a^	8.3	8.2	0.161	15.2	10.1	^a^	10.2	6.5	^a^	8.0	8.3	^b^
Seg.10	13.8	9.3	^a^	9.8	5.8	^a^	8.3	8.3	0.117	13.7	9.2	^a^	10.0	5.8	^a^	8.5	8.2	^b^
Seg.11	11.4	8.6	^a^	8.9	5.7	^a^	8.3	8.3	0.073	11.3	8.6	^a^	9.1	5.7	^a^	8.5	8.2	^b^
Seg.12	11.4	8.6	^a^	9.0	5.7	^a^	8.3	8.2	0.244	11.4	8.6	^a^	9.3	5.7	^a^	8.5	8.2	^b^

^a^ p<0.001

^b^ p<0.01

^c^ p<0.05

**Table 2:** Sensitivity, specificity and AUC of diagnostic classification test.

	*Conventional Model*			*Proposed Model*		
	*Sensitivity*	*Specificity*	*AUC*	*Sensitivity*	*Specificity*	*AUC*
Seg.1	0.85	0.26	0.27	0.58	0.71	0.72
Seg.2	0.60	0.34	0.57	0.52	0.31	0.64
Seg.3	0.59	0.20	0.61	0.46	0.34	0.68
Seg.4	0.83	0.29	0.55	0.68	0.80	0.79
Seg.5	0.85	0.31	0.63	0.69	0.74	0.77
Seg.6	0.85	0.31	0.63	0.64	0.80	0.75
Seg.7	0.96	0.14	0.65	0.58	0.66	0.67
Seg.8	0.85	0.31	0.71	0.43	0.34	0.65
Seg.9	0.87	0.31	0.55	0.44	0.34	0.65
Seg.10	0.96	0.29	0.66	0.56	0.69	0.67
Seg.11	0.94	0.31	0.75	0.58	0.69	0.68
Seg.12	0.96	0.20	0.65	0.57	0.69	0.66
Mean	0.84±0.12	0.27±0.06	0.60±0.12	0.56±0.08	0.59±0.19	0.69±0.05

The result of intraoperator and interoperator variability test showed the final result could be different, in Table 3. However, the difference between measured areas of each segment was not significant, except that of segment 2 for interoperator comparison with first operation (p‐value<0.05).

**Table 3:** Comparison of intraoperator and interoperator variability. Data are reported as mean values.

	*Operator A*			*Operator B*		
	*1st operation*	*2nd operation*	*Interoperator*	*1st operation*	*Intraoperator (wt. 1st op.)*	*Intraoperator (wt. 1st op.)*
*Segment*	*Area (cm^2^)*		p‐*value*	*Area (cm^2^)*	p‐*value*	
Seg.1	13.4±0.6	13.1±0.3	^b^	13.0±0.5	^b^	^b^
Seg.2	12.9±1.5	14.3±1.2	^b^	14.5±1.7	^a^	^b^
Seg.3	13.9±1.7	14.9±1.1	^b^	15.1±1.6	^b^	^b^
Seg.4	13.4±0.6	13.1±0.3	^b^	13.0±0.5	^b^	^b^
Seg.5	13.4±0.6	13.1±0.3	^b^	13.0±0.5	^b^	^b^
Seg.6	13.4±0.6	13.1±0.3	^b^	13.0±0.5	^b^	^b^
Seg.7	8.3±0.4	8.1±0.3	^b^	8.1±0.4	^b^	^b^
Seg.8	8.3±0.8	8.6±0.6	^b^	8.7±0.8	^b^	^b^
Seg.9	8.3±0.8	8.6±0.6	^b^	8.7±0.8	^b^	^b^
Seg.10	8.3±0.4	8.1±0.3	^b^	8.1±0.4	^b^	^b^
Seg.11	8.3±0.4	8.1±0.3	^b^	8.1±0.4	^b^	^b^
Seg.l2	8.3±0.4	8.1±0.3	^b^	8.1±0.4	^b^	^b^

^a^ p<0.05

^b^ p≥0.05

## IV. DISCUSSION

Our proposed model divided the LV myocardium into 17 segments because the 17‐segment model has used in routine clinical work since it was recommended by AHA and provides the best agreement with the available anatomic data and has the best fit with the methods commonly used in both echocardiography and SPECT nuclear cardiology.^(2)^ However, the proposed 17‐segment model considers the patient‐specific morphological variations in the LV anatomy to obtain a more precise mapping between segments and the AHA recommended nomenclature, while the conventional 17‐segment model divided the basal and midcavity slices into 6 segments of 60° each and the apical slices into four segments of 90° each. It is difficult to assign an individual segment to specific coronary artery territory in the conventional 17‐segment model because there is tremendous variability in the coronary artery blood supply to myocardial segments.

The proposed 17‐segment model divided the LV myocardium by considering the position of two interventricular grooves in the circumferential location. These grooves were recommended to separate and identify the septum from the left ventricular anterior and inferior free walls by the AHA.^(2)^ However, the conventional model divided the basal and midcavity slices into 6 segments of 60° each and the apical slices into four segments of 90° each because it is a standardized model, and thus has difficulties in reflecting anatomic variability. The boundaries of the LV septum region, the two interventricular grooves, were overlaid on top of the bull's‐eye map. The patient‐specific bull's‐eye map shows different segmental areas based on patients' anatomic variations. To validate the proposed model, the severe AS patient group was chosen because LV geometry analysis for AS is related not to the supplying coronary artery of each segment, but to morphology and location. AS is a prototypical example of LV hypertrophy because pressure overloading can change the LV geometry in patients with normal coronary arteries.^(27)^ This characteristic of AS seemed to be suitable for validating the usability and clinical value of the proposed model.

Even though we could have expected this result from the segmental area distribution of the proposed model shown in Fig. 6(d), this is the first evidence showing that the segmental area could be used for differentiating severe AS patients from normal controls. Figure 6(d) indicates that each segmental area of severe AS patients did not overlap with that of normal controls, whereas the areas of the two groups were almost the same, except for segments 3, 4, and 5 of the conventional model (Fig. 6(a)). The conventional model divided basal slices into six segments of 60° each; however, the area of segments 2–4 in Fig. 6(a) differed from each other because the aortic root was excluded from LV volume, as described in the Materials and Methods section B. As shown in Figs. 6(b), 6(c), 6(e), and 6(f), the results of the two models were very similar and the areas of the conventional bull's‐eye map show no patient‐specific anatomical variations. Regarding the recommended nomenclature view, segment 2 in the left anterior descending coronary artery territory and segment 3 in the right coronary artery territory in the conventional bull's‐eye map could not be as clearly mapped to the LV septum region as their nomenclature (i.e., basal anteroseptal and basal inferoseptal, respectively). In contrast, the patient‐specific bull's‐eye map showed morphological boundaries that were projected from the 3D boundaries by considering patient‐specific morphological variations. Therefore, our method could help to understand the LV morphology at a glance and to minimize the gap between the 3D and 2D LV morphologies.

In clinical purposes, the 2D bull's‐eye map is preferable due to understanding of patient variation at a glance, even though the 3D display would give more accurate and informative patients' information. In addition, medical doctors have used the 2D bull's‐eye map in routine clinical work. Therefore, it is very important to visualize the 2D bull's‐eye map based on the patient‐specific 17‐segment model. The use of patient‐specific segmental areas on the bull's‐eye map could simultaneously increase physician recognition of a patient's specific LV anatomical variations and provide a functional map that includes LV thickness and an intensity map of perfusion, along with other parameters. In addition, we evaluated a correlation between patient weight and our measurements. But we could not find a correspondence between them. However, further study of interrelationships may be helpful in finding a hidden relationship between our measurements and other clinical indexes.

The color coding in Figs. 3(e), and 3(k) include thin segments in both panels, and a wedge‐shaped segment in 3(k) because the shape of LV myocardium is not a circular cone and the centerline of LV myocardium is not a line, as depicted in Fig. 4(a). If the centerline is a straight line, the both models do not include thin segments. But the proposed model will include a wedge‐shaped segment because the two interventricular grooves were rotated when moving toward the base of the heart from the apex (Figs. 3(g), 3(h), 3(i), and 3(j)).

The differentiating capability of the proposed model was evaluated by performing a diagnostic classification test using the maximum likelihood that was estimated from the area of each segment (Table 1). The sensitivity was decreased, but the specificity and AUC were increased in the proposed model. It might be due to the different size of each segmental area. The area of each segment in the proposed model was different in size, and distribution shape was similar to a gentle bell curve, while that in the conventional model showed less variation and its distribution shape was similar to a sharp bell curve. Thus, the estimated maximum likelihood caused lower sensitivity and higher specificity and AUC in the proposed model than in the conventional model. There were 4 segments where the AUC was bigger than 0.75. The AUC of segment 11 for the conventional model was 0.75, but the sensitivity and specificity were not balanced (sensitivity: 0.94 and specificity: 0.31). However, the AUC of segments 4, 5, and 6 for the proposed model was bigger than 0.75 and the sensitivity and specificity were balanced in comparison to the conventional model (sensitivity>0.64 and specificity>0.74). Figure 6(d) and Table 1 also show that the area of segments 4, 5, and 6 have enough difference to divide the two groups without any optimization. This evaluation result shows a possibility that using a part of the 2D segmental area in this proposed model can be useful for the diagnosis of severe AS by using machine learning.

This study had several limitations. First, our method required manual adjustment at each step. Therefore, the final result could be affected by manual delineation of surface contours (Table 3). An automatic alignment method of 17 myocardial segments should be developed in the future to increase the accuracy and reproducibility. In addition, the LV segmentation method should be enhanced in the future. The already segmented 179 LV volumes will be good input data for multi‐atlas‐based LV segmentation. Second, we directly compared the proposed model to the AHA model using all segmented short axis slices in which there are 360° of myocardium. It allows more accurate and precise evaluation because our method measured not on short axis slice but on the 3D LV volume. However, it differs from the AHA guidelines for selection and thickness of cardiac slices for display. Finally, our validation was based on a limited number of CT scans and was performed in select patients with severe AS and in normal controls. To increase usability, the method should be validated in further studies that include a greater number of all‐comer patients with various types and severities of disease.

## V. CONCLUSIONS

The conventional myocardial segmentation model has been used to date for quantitative analysis of the LV. However, it does not consider patient‐specific myocardial anatomic variations. We here propose a patient‐specific, 17‐segment myocardial model based on a quantitative cardiac analysis platform. The 2D and 3D quantitative parameters generated by this platform can be used to differentiate between normal controls and severe AS patients. In particular, the differentiating capability of both 2D and 3D parameters of the proposed model demonstrates statistically significant differences (all p‐values<0.011). The patient‐specific bull's‐eye map will help to understand the LV morphology at a glance and to minimize the gap between 3D and 2D LV morphologies because it simultaneously represents patient‐specific segment information on the map with visualization of morphological boundaries. It is therefore superior to the conventional 17‐segment model.

## ACKNOWLEDGMENTS

This research was supported by the Basic Science Research Program through the National Research Foundation of Korea, funded by the Ministry of Science, ICT, and Future Planning (NRF‐2013R1A1A1058711). This research was supported by a grant of the Korea Health Technology R&D Project through the Korea Health Industry Development Institute (KHIDI), funded by the Ministry of Health & Welfare, Republic of Korea (HI14C0517, HI12C0630). The authors especially thank Youngsin Kim, Songyi Baek, and Jiyeun Ko for helping with the experimental work.

## COPYRIGHT

This work is licensed under a Creative Commons Attribution 3.0 Unported License.
